# Idiopathic posterior papillary muscle rupture; a case report

**DOI:** 10.1186/s12872-022-02570-4

**Published:** 2022-04-06

**Authors:** Ioannis Milioglou, Matthew R. Janko, Hafeez ul Hassan, Mohammed ElHaq, Steven J. Filby, Marc P. Pelletier

**Affiliations:** 1 Departments of Cardiology, University Hospitals Cleveland Medical Center, 11100 Euclid Ave, Cleveland, OH 44106 USA; 2 Departments of Cardiac Surgery, University Hospitals Cleveland Medical Center, 11100 Euclid Ave, Cleveland, OH 44106 USA; 3 Departments of Pathology, University Hospitals Cleveland Medical Center, 11100 Euclid Ave, Cleveland, OH 44106 USA

**Keywords:** Papillary muscles, Mitral valve, Shortness of breath, Valve repair

## Abstract

**Background:**

Papillary muscle rupture is a rare condition. Its clinical presentation, diagnosis and management can be very challenging for the clinician.

**Case presentation:**

A 73-year-old woman with hypertension presented with chest pain, ST-segment changes, and elevated serum troponin levels. Coronary angiography was normal. Echocardiography revealed normal ventricular function, flail posterior mitral leaflet, and severe mitral regurgitation. She underwent emergent mitral valve replacement.

**Conclusion:**

The diagnostic and management strategies of this uncommon presentation are discussed.

**Supplementary Information:**

The online version contains supplementary material available at 10.1186/s12872-022-02570-4.

## Background

Papillary muscle rupture (PMR) is itself uncommon, with incidence rates of 0.029% in patients presenting with MI, and few case reports of non-MI etiology [1]. Compared to patients without PMR, those with PMR had significantly higher in-hospital mortality rates (5.3% vs 36.3%, p < 0.001) [1]. Papillary muscle rupture usually occurs within 1 week post infraction. These patients present acutely with signs of acute hypoxemic respiratory failure due to severe pulmonary edema. Hypotension secondary to cardiogenic shock has also been reported. Urgent surgery is the recommended approach for such patients given the high mortality rate of this condition [2].

## Timeline

**Table:** 

Emergency department	Chest Pain + ST depressions II-III-AVF, V4-V6, ST elevation in aVR + positive troponins, Cath Lab Activation
Cath lab	-No evidence of coronary arterial disease -Ventriculogram and subsequent ECHO indicative of severe mitral regurgitation -IABP placement and intubation
Cardiology intensive unit	Patient diuresed and stabilized prior to surgery
Surgery	-Posterior papillary muscle rupture -Mitral valve replacement with bioprsethetic Epic 31 mm
Cadiothoracic surgery intensive care unit	Patient weaned off IABP and inotropes

## Case presentation

A 73-year-old woman with a medical history of hypertension presented to the emergency department after 30 min of severe pressure-like substernal chest pain that radiated to her left arm and jaw at rest. The pain began 30 min prior to presentation. Her review of systems was positive for nausea and acute shortness of breath at rest. Pertinent negatives included any history of these symptoms, dyspnea on exertion at baseline, absence of trauma, initiation of new medications, exposure to sick contacts, or recent travel.

Patient's past medical history was significant for hypertension, generalized anxiety disorder, major depressive disorder, hypertension, and severe osteoarthritis. Patient was noted to have atypical polymyalgia rheumatica for which she was placed on 6 months taper course of steroids. She has been under the care of rheumatology with autoimmune and inflammatory markers all negative, indicative of osteoarthritis. Patient was on buspirone, duloxetine, hydrochlorothiazide, valsartan and meloxicam as needed. Patient's family history was significant for a father with sudden cardiac death due to dilated cardiomyopathy. No other risk factors or high-risk behaviors were reported.

On physical examination, the patient was normothermic and tachycardic with a rate of 112 beats per minute. Her blood pressure was 88/55 mm of Mercury (mmHg) and peripheral oxygen saturation was 88% on 5 L of supplemental oxygen via nasal cannula. She was in acute distress, diaphoretic, with jugular venous distension to the mid-neck, and bibasilar crackles. Normal S1 and decreased S2 were noted, with a holosystolic murmur radiating to her axilla; well-perfused extremities with + 1 lower extremity edema to the knees were also noted.

**Fig. 1:**
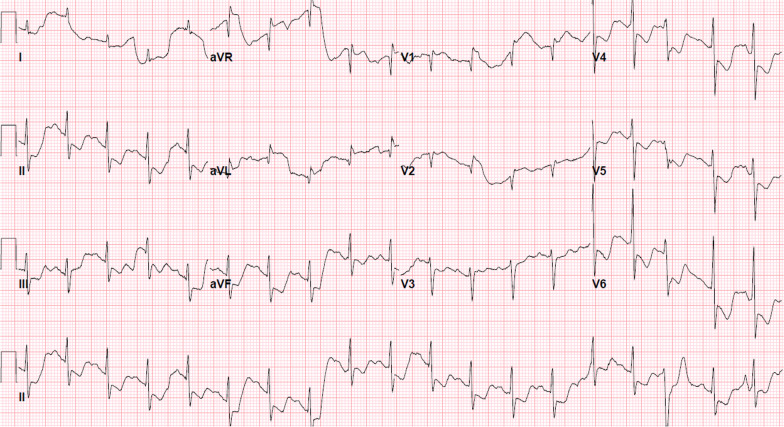
12 lead Electrocardiogram on Admission; sinus tachycardia BPM 114, normal intervals and axis, diffuse ST depressions II-III-AVF, V4-V6, ST elevation in aVR

Initial electrocardiogram was indicative of diffuse ST-segment depressions and ST-segment elevation in lead aVR (Fig. 1). Initial blood investigations were notable for lactate of 1.7 mg/dl and troponin of 1.33 ng/dL. She was given aspirin, ticagrelor, and heparin infusion in the emergency department. Catheter angiography was negative for coronary artery disease with less than 30% of obstruction of all coronary vessels (Additional file 1 and 2: Videos 1 & 2). Patient's left ventricular end-diastolic pressure was elevated at 31 mmHg and left ventriculopgraphy was indicative of severe (4 +) mitral regurgitation (MR) with dilated left atrium (Additional file 3: Video 3). Transthoracic echocardiogram (TTE) in the cath lab showed a left ventricular ejection fraction (LVEF) of 65%, severe MR, flail posterior mitral valve leaflet as well as flail head of posterior papillary muscle (Additional file 4: Video 4).

**Fig. 2:**
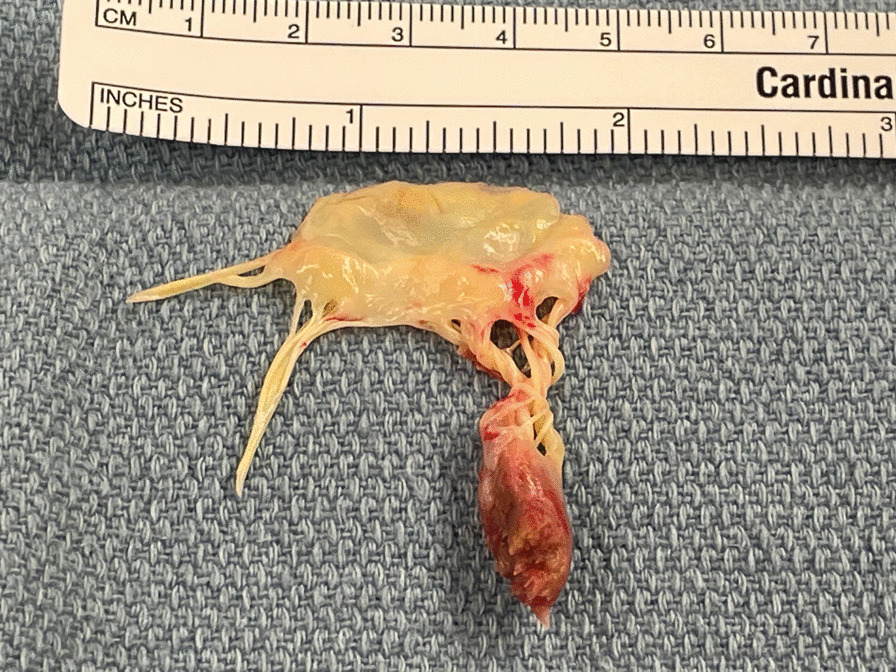
Macroscopic specimen figure, ruptured papillary muscle head along with posteromedial mitral valve leaflet

**Fig. 3:**
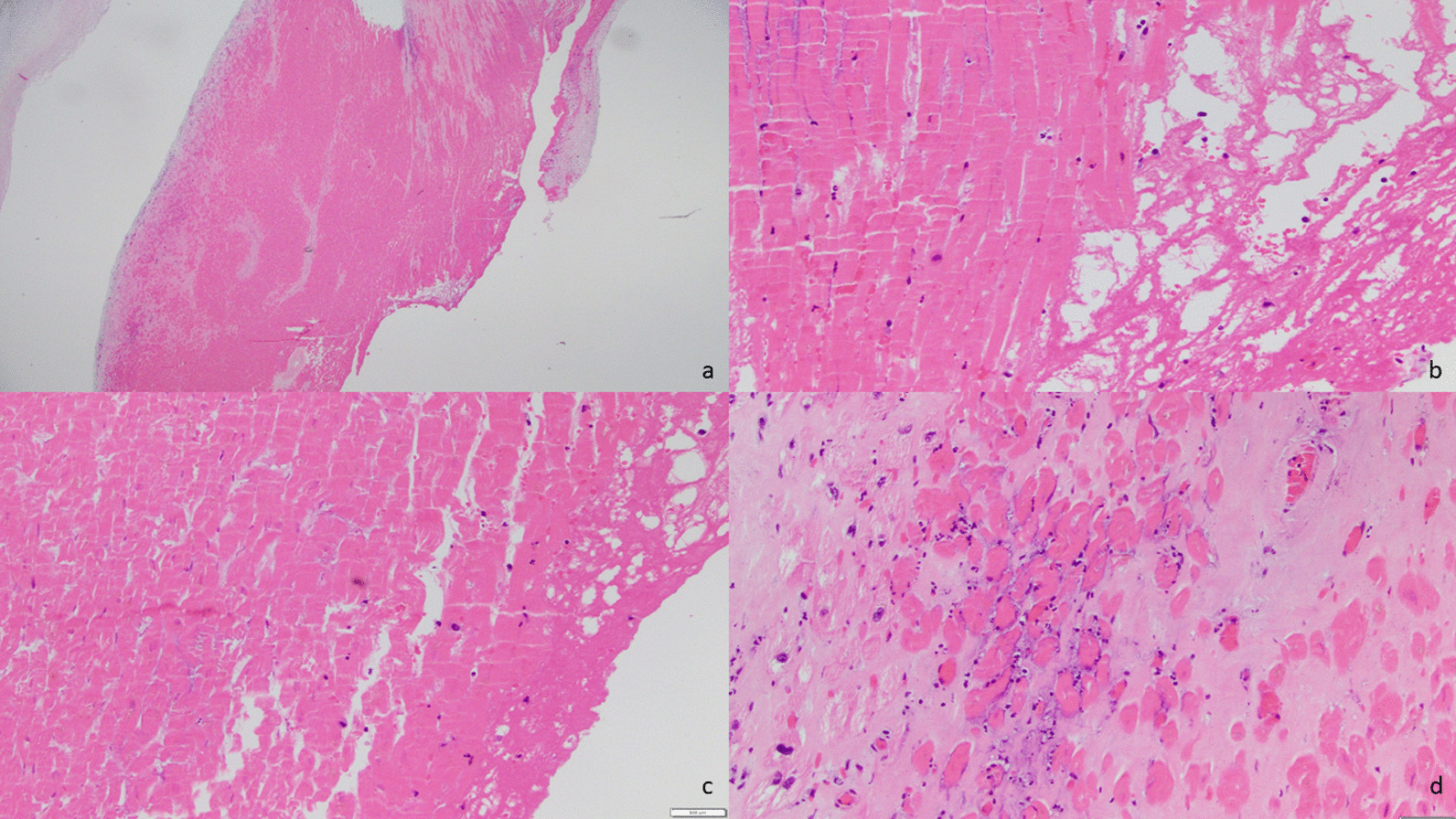
**a** Low power image (1.25×) showing the resected papillary muscle. **b**, **c** Higher power images show eosinophilic myocytes consistent with myocyte necrosis, and adjacent hemorrhage. **d** A higher power image demonstrating myocyte necrosis with associated neutrophilic infiltration

An intra-aortic balloon pump (IABP) was placed in the cardiac catheterization laboratory and patient was admitted to the cardiac intensive unit for diuresis. She was intubated in the cath lab due to severe acute hypoxemic respiratory failure. She improved with diuresis and hemodynamic support. She was taken for operative repair on hospital day 3. Intraoperative transesophageal echocardiogram (TEE) revealed severe eccentric MR (Additional file 5: Video 5). After standard initiation of cardiopulmonary bypass and cardiac arrest, direct visual inspection revealed a hemorrhagic posteromedial papillary muscle. The decision was made to replace the irreparably injured valve, and a 31 mm Epic™ bioprosthetic valve was placed. Postoperative TEE showed no evidence of residual MR and mean gradient of 2 mmHg (Additional file 6: Video 6). Pathologic specimen revealed ruptured posterior papillary muscle head, without any vegetations, calcifications, perforations; chordae tendinae appeared normal (Fig. 2). Microscopic pathologic evaluation revealed ruptured papillary muscle with myocyte necrosis and hemorrhage secondary to the rupture (Figs. 3a–d).

Patient was diagnosed with acute severe mitral valve regurgitation secondary to posterior papillary muscle rupture (PMR). Given patient's presentation consistent with acute myocardial ischemia, myocardial infarction (MI) complicated by PMR was higher in our differential. Nonetheless, coronary angiogram showed no evidence of coronary artery disease. Coronary spasm, myocardial infraction without coronary artery occlusion (MINOCA) or acute coronary artery thrombosis followed by spontaneous recanalization could not be ruled out at this point. The patient’s unstable hemodynamic status did not allow for a magnetic resonance imaging (MRI) prior to surgery. A diagnosis of idiopathic papillary muscle rupture leading to severe acute mitral regurgitation was made.

The patient had a prolonged postoperative hospitalization complicated by respiratory failure and ventricular cataplexy requiring prolonged support with IABP and milrinone. Cardiac output and cardiac index did improve to normal, she was weaned off all support and was discharged from the hospital. There was no MR or decreased LVEF on follow up transthoracic echo.

## Discussion and conclusion

We present a rare case of idiopathic papillary muscle rupture causing severe MR requiring urgent surgical correction with good outcome. Spontaneous PMR in the absence of coronary artery stenosis is rarely reported in the literature [3–5], however, it has been associated with endocarditis, blunt chest trauma, Takotsubo cardiomyopathy, and acute pancreatitis [6, 7]. Coronary arterial spasm manifesting as ischemia has also been reported with PMR in a single case report [8]. Nonetheless, myocardial ischemia is the most common mechanism of reported PMR. The papillary muscles are highly vulnerable to damage induced by hypoxia or ischemia, and the posterior papillary muscle is most commonly affected given its typically poorly-collateralized arterial supply by branches of right posterior descending or right posterolateral artery or the left coronary obtuse marginal arteries [9]. The coronary angiogram of our patient did not demonstrate any vessel obstruction or evidence of coronary artery disease. Myocardial infarction without coronary artery occlusion or chronic microvascular ischemia could be plausible etiologies for our patient as demonstrated in other case reports, albeit unlikely given the lack of chronic ischemic symptoms in our patient’s history [3]. The vulnerability of the papillary muscle is emphasized by the fact that recent or old papillary muscle infarction were detected in 25% of autopsy cases [10]. Cardiac MRI is a useful tool in differentiating ischemic tissue from other processes; unfortunately the acuity of our case did not allow for this study.

Initial medical management of PMR includes diuretics, and oxygenation delivered with non-invasive and invasive mechanical ventilation. Mechanical support with IABP should be considered in patients presenting with cardiogenic shock [2]. Nevertheless, prompt diagnosis and early surgical management are the cornerstones of treatment. Mortality rates stemming of retrospective surgical cohorts in the past 2 decades are between 25 to 30% (30 day post-operatively) [11]. Current data on catheter-based edge-to-edge techniques for this clinical entity are not robust, with few case reports in the literature [11].

Acute mitral regurgitation as a complication of myocardial infraction has a very poor prognosis if left untreated. We present a rare case of idiopathic papillary muscle rupture causing severe MR requiring urgent surgical correction with good outcome.

## Supplementary Information

**Additional file 1: Video 1.** RAO CRAN view of Right Coronary Artery; Less than 30% occlusion

**Additional file 2: Video 2.** RAO Caudal view of Left Main, Circumflex and Anterior Descending Arteries; less than 30% occlusion in all territories

**Additional file 3: Video 3.** Ventriculogram of left ventricle showing + 4 regurgitation jet in a severely dilated left atrium

**Additional file 4: Video 4.** Posterior Long Axis View, Transthoracic ECHO; Flail posteromedial mitral valve cusp, eccentric regurgitant jet, dilated left atrium

**Additional file 5: Video 5.** Transesophageal ECHO, midesophageal 3 chamber view; dilated LA, eccentric regurgitant jet, vena contracta 0.6 cm

**Additional file 6: Video 6.** Intraoperative Transesophageal ECHO Post Mitral Valve Replacement, midesophageal 4 chamber view; 31 mm Epic bioprosthetic valve, no regurgitation

## Data Availability

The datasets used and/or analysed during the current study are available from the corresponding author on reasonable request.

## Abbreviations

MI: Myocardial infarction
PMR: Papillary muscle rupture
MINOCA: Myocardial infraction without coronary artery occlusion
MR: Mitral regurgitation
TTE: Transthoracic echocardiogram

## References

[1] Bhardwaj B (2020). Outcomes and hospital utilization in patients with papillary muscle rupture associated with acute myocardial infarction. Am J Cardiol.

[2] Burton LV, Beier K. Papillary Muscle Rupture. [Updated 2020 Aug 24]. In: StatPearls [Internet]. Treasure Island (FL): StatPearls Publishing; 2021 Jan. https://www.ncbi.nlm.nih.gov/books/NBK499976/29763151

[3] Sakata T (2017). Rupture of anterolateral papillary muscle resulting from small artery occlusion. Ann Thorac Surg.

[4] Shim, C.Y. et al. Spontaneous rupture of a papillary muscle. Circulation 127 (2013) 10.1161/CIRCULATIONAHA.112.142448 23648683

[5] Lee H (2009). New-onset heart failure caused by spontaneous papillary muscle rupture: diagnosis with dual-source computed tomographic coronary angiography. J Thorac Cardiovasc Surg.

[6] Ballo P, Mangialavori G, Betti I, Giunti G, Meucci F, Chiodi L et al. Isolated papillary muscle rupture complicating acute pancreatitis. Ann Thorac Surg 36–38 (2011). 10.1016/j.athoracsur.2010.10.067 21352966

[7] Yaghoubi AR, Ansarin K, Hashemzadeh S, Azhough R, Faraji S, Bozorgi F. Tako-tsubo cardiomyopathy induced by emotional stress leading to severe mitral regurgitation, cardiogenic shock and cardiopulmonary arrest. Int J Cardiol 85–86 (2009). 10.1016/j.ijcard.2008.04.047 18657330

[8] Yamazaki M, Fukui T, Mahara K, Takanashi S (2015). Complete rupture of the anterolateral papillary muscle caused by coronary spasm. Interact Cardiovasc Thorac Surg.

[9] James TN (1965). Anatomy of the coronary arteries in health and disease. Circulation.

[10] DePasquale NP, Burch GE (1966). The necropsy incidence of gross scars or acute infarction of the papillary muscles of the left ventricle. Am J Cardiol.

[11] Valle, J. A., Miyasaka, R. L. & Carroll, J. D. Acute mitral regurgitation secondary to papillary muscle tear: is transcatheter edge to-edge mitral valve repair a new paradigm? Circ Cardiovasc Interv. 10 (2017). 10.1161/CIRCINTERVENTIONS.117.005050 28607001

